# Glofitamab in the sequential treatment of relapsed/refractory B-Cell lymphoma: a single-center real-world study

**DOI:** 10.3389/fonc.2026.1885982

**Published:** 2026-07-07

**Authors:** Haoran Xu, Fangce Wang, Chenbo Sun, Bing Xiu, Yi Ding, Bin Xue, Aibin Liang, Wenjun Zhang

**Affiliations:** 1 Department of Hematology, Shanghai Tongji Hospital, Tongji University School of Medicine, Shanghai, China; 2 Clinical Research Ward of Cancer Center, Shanghai Tongji Hospital, Tongji University School of Medicine, Shanghai, China

**Keywords:** bispecific antibody, CAR-T therapy, Glofitamab, relapsed/refractory B-cell lymphoma, sequential treatment

## Abstract

**Background:**

Glofitamab is a bispecific antibody (BsAb), and has good efficacy in relapsed or refractory B-cell non-Hodgkin lymphoma (r/r B-NHL), especially in relapsed/refractory diffuse large B-cell lymphoma (r/r DLBCL). This study explored its sequential treatment strategies and efficacy in the real world, including rescue therapy after chimeric antigen receptor T cell (CAR-T) therapy failure, bridging CAR-T therapy and monotherapy.

**Methods:**

This was a single-center retrospective cohort analysis of 24 patients with clearly diagnosed relapsed/refractory B-cell lymphoma, including primary diffuse large B-cell lymphoma (DLBCL), diffuse large B-cell lymphoma with Richter transformation (DLBCL-RT), and Burkitt lymphoma (BL). Patients were divided into three groups according to the treatment sequence: Group 1 (Glofitamab rescue therapy after progression of CAR-T therapy), group 2 (Glofitamab bridging subsequent CAR-T therapy), and group 3 (Glofitamab monotherapy), who received Glofitamab at our center between September 2024 and December 2025.

**Conclusions:**

In the single-center real-world experience, Glofitamab demonstrated controllable safety and durable anti-tumor activity in three clinical scenarios. Especially, it could still bring clinical benefits even after CAR-T therapy failed, and it explored the sequential treatment strategy of Glofitamab and CAR-T.

## 1 Introduction

Diffuse large B-cell lymphoma (DLBCL) is the most common form of aggressive non-Hodgkin lymphoma (NHL), making up about 30% of all NHL (1, 2). At present, the standard treatment for DLBCL patients is rituximab plus cyclophosphamide, doxorubicin, vincristine and prednisone (R-CHOP), but about 30% to 50% of patients cannot be cured. In addition, 30% of patients relapse after achieving complete remission (CR) after treatment (3). Diffuse large B-cell lymphoma with Richter transformation (DLBCL-RT) refers to the histologic evolution of chronic lymphocytic leukemia (CLL) to DLBCL. This is a transformation that is seen in about 2-10% of CLL patients. The prognosis is usually bad, and median overall survival (OS) is reported to be only between 6 and 12 months (4, 5). Burkitt lymphoma (BL) is another highly aggressive NHL derived from B lymphocytes, which is rapidly growing and sensitive to chemotherapy. The current treatment regimen is high-dose chemotherapy based on rituximab, but some patients cannot tolerate the intensity of treatment and have a poor prognosis (6, 7). For the patients, who have relapsed or refractory B-cell lymphoma (r/r BCL), the variety of treatment strategies is limited. Therefore, it is important to explore other and effective therapeutic ways.

Bispecific antibody (BsAb) are one of the new ways in immunotherapy and provide new strategies to improve the management of hematological cancers, especially for the cases of relapsed or refractory B-cell non-Hodgkin lymphoma(r/r B-NHL) (8, 9). Glofitamab, a novel CD20×CD3 BsAbs with a unique 2:1 T cell binding structure, has shown promising efficacy as a rescue therapy for r/r B-NHL (10, 11). One study demonstrated significant efficacy of Glofitamab in patients with relapsed/refractory diffuse large B-cell lymphoma (r/r DLBCL), with 39% of patients achieving CR at a median follow-up of 12.6 months and a 12-month progression-free survival rate of 37% (11). Another study of evaluating the glofitamab in combination of gemcitabine-oxaliplatin have showed a mark improvement of overall survival for the r/r DLBCL patients. Cytokine release syndrome (CRS) occurred in 44% of patients and was mostly low-grade (12).In addition, some studies have shown that Glofitamab also has a good therapeutic effect on patients with DLBCL-RT (13).All these show that Glofitamab is an important therapeutic approaches for r/r B-NHL.

Compared with BsAbs, chimeric antigen receptor T cell (CAR-T) is superior to BsAbs in improving CR rate. However, BsAbs are less likely to experience CRS, Immune Effector Cell-Associated Neurotoxicity Syndrome (ICANS), and infection-related adverse events of grade ≥3, and their treatment can be appropriately adjusted by controlling dose levels. It is helpful for the management and prevention of adverse events (14, 15).BsAb can be used as a bridge to alternative radiotherapy and chemotherapy until CAR-T infusion is prepared, and as a subsequent salvage maintenance therapy for patients who do not respond to CAR-T therapy or who are reviewed. Given the complementary mechanisms of action of the two in r/r B-NHL, BsAb can be used as an alternative to radiotherapy and chemotherapy before CAR-T infusion is prepared. The combination of CAR T and Glofitamab has been explored in several studies. Furthermore, studies have shown that the combination of the two treatment regimens is superior to either regimen alone (16–20). However, the optimal treatment sequence for CAR T and Glofitamab is still unclear, and their application patterns and efficacy data in real-world clinical practice remain to be fully elucidated (21), especially in three scenarios with different clinical needs: (1) rescue therapy after CAR-T therapy failure; (2) Bridging to CAR-T therapy; (3) Glofitamab is not suitable for CAR-T monotherapy, and the efficacy and safety of Glofitamab have not been systematically compared.

## 2 Materials and methods

### 2.1 Patients

This study was a single-center retrospective cohort study that consecutively enrolled 24 adult patients (≥18 years old) with r/r LBCL who were treated with Glofitamab at our center between September 2024 and December 2025. All patients had a pathological diagnosis of LBCL and had received at least two lines of systemic therapy. According to treatment intention and sequence, patients were divided into three groups: Group 1 (Glofitamab as rescue therapy after failure of CAR-T therapy), Group 2 (Glofitamab as bridging therapy to sequential CAR-T therapy after disease control), and Group 3 (Glofitamab monotherapy, without bridging and not after CAR-T therapy).The study involving human participants were reviewed and approved bythe Institutional Ethics Committee of our hospital. The patients/participants provided their written informed consent to participate in this study.

### 2.2 Treatment and assessment

Glofitamab followed a step-up dosing study regimen: all patients received a single dose of Obinutuzumab 1000 mg intravenously on day 1 of cycle 1 (D0) to deplete peripheral B cells (except for one patient who did not used due to severe baseline B cell insufficiency). Patients in groups 1 and 3 received 2.5 mg on day 8 (D7) of cycle 1 and 10 mg on day 15 (D14), followed by a fixed dose of 30 mg every 3 weeks from cycle 2 onwards until completion of 12 cycles, disease progression, or unacceptable toxicity (11). Patients in group 2 received a planned cycle of Glofitamab bridging therapy with 2.5 mg on day 8 (D7), 10 mg on day 15 (D14), and 30mg on day 22 (D21) on cycle 1, followed by CD19 CAR-T cell infusion as scheduled.

Efficacy was evaluated by imaging at baseline, regularly during treatment, and according to the clinical judgment of the treating physician, and divided into CR, partial response (PR), and progressive disease (PD), all based on the Lugano 2014 criteria (22). CRS and ICANS were graded according to the 2019 ASTCT consensus criteria (23). Immune effector cell-associated hemophagocytic lymphohistiocytosis like syndrome (IEC-HS) was evaluated according to the 2023 ASTCT consensus criteria (24).In addition, safety data on hematological adverse events, infections, and liver or kidney injury were classified on the basis of CTCAE criteria, version 5.0. The absolute counts of CD4+ T cells, CD8+ T cells and NK cells in peripheral blood of patients before and after treatment were detected by flow cytometry to analyze the dynamic of immune cells.

### 2.3 Statistical analysis

The primary efficacy endpoint was progression-free survival (PFS), defined differently according to the group: group 2 (bridging group) was calculated from the date of CAR-T cell infusion until the occurrence of an event (disease progression or death from any cause); Secondary end points included overall response rate (ORR) and complete response rate (CRR), calculated from the date of first Glofitamab dose. PFS, ORR, and CRR were estimated by the Kaplan-Meier method and are reported as medians with 95% confidence intervals (CI). Statistical analyses were performed using the survival package in R, and patient characteristics were tested by χ2 to compare differences between groups. A p value of less than 0.05 was considered to indicate statistical significance.

## 3 Results

### 3.1 Patient characteristics

A total of 24 patients with r/r BCL treated with Glofitamab were enrolled in this study, which were divided into three groups according to the treatment regimen: There were 7 patients in group 1 (Glofitamab as rescue therapy after the failure of CAR-T therapy), 10 patients in group 2 (Glofitamab bridging CAR-T therapy), and 7 patients in group 3 (Glofitamab monotherapy). The baseline characteristics of all the patients are shown in **Table 1**. There was no significant difference in age, gender, Ann Arbor stage, international prognostic index (IPI) score, other concurrent treatment regimens and previous lines of treatment among the three groups (all p>0.05). The median age of the three groups was 61 years (range 27–70 years), 60 years (range 25–71 years) and 62 years (range 42–83 years), respectively, and the proportion of women was 28.6%, 20.0% and 14.3%, respectively. The median IPI score was 3 (range 2-4), 3 (range 2-5) and 4 (range 2-4), respectively. In terms of disease types, *De novo* DLBCL was the main type of DLBCL in group 1 (71.4%), *De novo* DLBCL accounted for 30.0% and DLBCL-Richter syndrome accounted for 30.0% in group 2, and *De novo* DLBCL accounted for 57.1% in group 3. TP53 mutation was detected by FISH in 1 case (14.3%), 4 cases (40.0%) and 0 case in group 1, 2 and 3, respectively. MYC/BCL6 rearrangement was found in 1 case in each of the three groups. MYC/BCL2 rearrangement was only found in group 2 (1 case) and group 3 (1 case). There were 71.4%, 40.0% and 71.4% of the patients without the above abnormalities, respectively. According to Ann Arbor staging, all patients in group 1 were in stage IV (IV A 57.1%, IV B 42.9%), 90.0% in group 2 were in stage IV A, and 85.7% in group 3 were in stage IV B. The median number of previous lines of therapy was 4 (range 2-8), 3 (range 1-5) and 2 (range 2-3), respectively. There were 3 cases (42.9%), 1 case (10.0%) and 0 cases with autologous transplantation history, respectively. In group 1, 4 patients (57.1%) did not receive bridging therapy, and the rest received Pola, BOL or ICE regimens. In group 2, there were 5 cases (50.0%) without bridging, and the rest received Pola, BOL or GemOx. In group 3, 6 patients (85.7%) did not receive bridging, and only 1 patient received GemOx.

**Table 1:** Baseline characteristics of patients with relapsed/refractory B-cell lymphoma treated with Glofitamab across three sequential treatment scenarios (Group 1, rescue therapy after CAR-T failure; Group 2, bridging to CAR-T; Group 3, monotherapy).

Characteristics		Treatment group 1 (rescue therapy after CAR-T failure; N=7)	Treatment group 2 (bridging to CAR-T; N=10)	Treatment group 3 (glofitamab monotherapy; N=7)
Age, Median(range)		61(27-70)	60(25-71)	62(42-83)
Gender - Female(N,%)		2(28.6%)	2(20.0%)	1(14.3%)
IPI score, Median(range)		3(2-4)	3(2-5)	4(2-4)
Disease type, N(%)				
	*De novo* DLBCL	5(71.4%)	3(30.0%)	4(57.1%)
	HGBL	1(14.3%)	2(20.0%)	2(28.6%)
	Burkitt	1(14.3%)	2(20.0%)	0(0%)
	DLBCL-Richter syndrome	0(0%)	3(30.0%)	1(14.3%)
FISH, N(%)				
	TP53 mutation	1(14.3%)	4(40.0%)	0(0%)
	MYC and BCL6 rearrangement	1(14.3%)	1(10.0%)	1(14.3%)
	MYC and BCL2 rearrangement	0(0%)	1(10.0%)	1(14.3%)
	None	5(71.4%)	4(40.0%)	5(71.4%)
Ann Arbor stage, N(%)				
	IV A	4(57.1%)	9(90.0%)	1(14.3%)
	IV B	3(42.9%)	1(10.0%)	6(85.7%)
Prior lines, Median(range)		4(2-8)	3(1-5)	2(2-3)
Prior autologous transplant, N(%)		3(42.9%)	1(10.0%)	0(0%)
Pre-glofitamab pharmacotherapy, N(%)				
	Pola	1(14.3%)	2(20.0%)	0(0%)
	BOL	1(14.3%)	2(20.0%)	0(0%)
	GemOx	0(0%)	1(10.0%)	1(14.3%)
	ICE	1(14.3%)	0(0%)	0(0%)
	None	4(57.1%)	5(50.0%)	6(85.7%)

No significant differences were observed among groups (all p>0.05). IPI,International Prognostic Index; *De novo* DLBC,*De novo* diffuse large B-cell lymphoma;HGBL,High-grade B-cell lymphoma; FISH,Fluorescence *In Situ* Hybridization;Pola,Polatuzumab Vedotin;BOL,BTK inhibitor (e.g., Zanubrutinib) + Obinutuzumab + Lenalidomide;GemOx,Gemcitabine + Oxaliplatin;ICE,Ifosfamide + Carboplatin + Etoposide.

In group 1, the median interval between the last previous CAR-T treatment and the current Glofitamab treatment was 126 days (range 46–219 days). In this group, there were two patients, which received two or more prior CAR-T. One patient did not receive Obinutuzumab because of big deficiency of B-cells in their baseline. In group 2, the median interval from CAR-T to Glofitamab completion was 34 days (range 20–90 days), with 1 patient (10.0%) undergoing concurrent autologous hematopoietic stem cell transplantation (auto-HSCT) 5 days prior to subsequent bridging CAR-T therapy. In group 3, one patient had break of Glofitamab because of severe pneumonia. All other patients in this group got the treatment according to the described treatment scheme in the above regimen.

### 3.2 Response and survival outcomes

**Figure 1:**
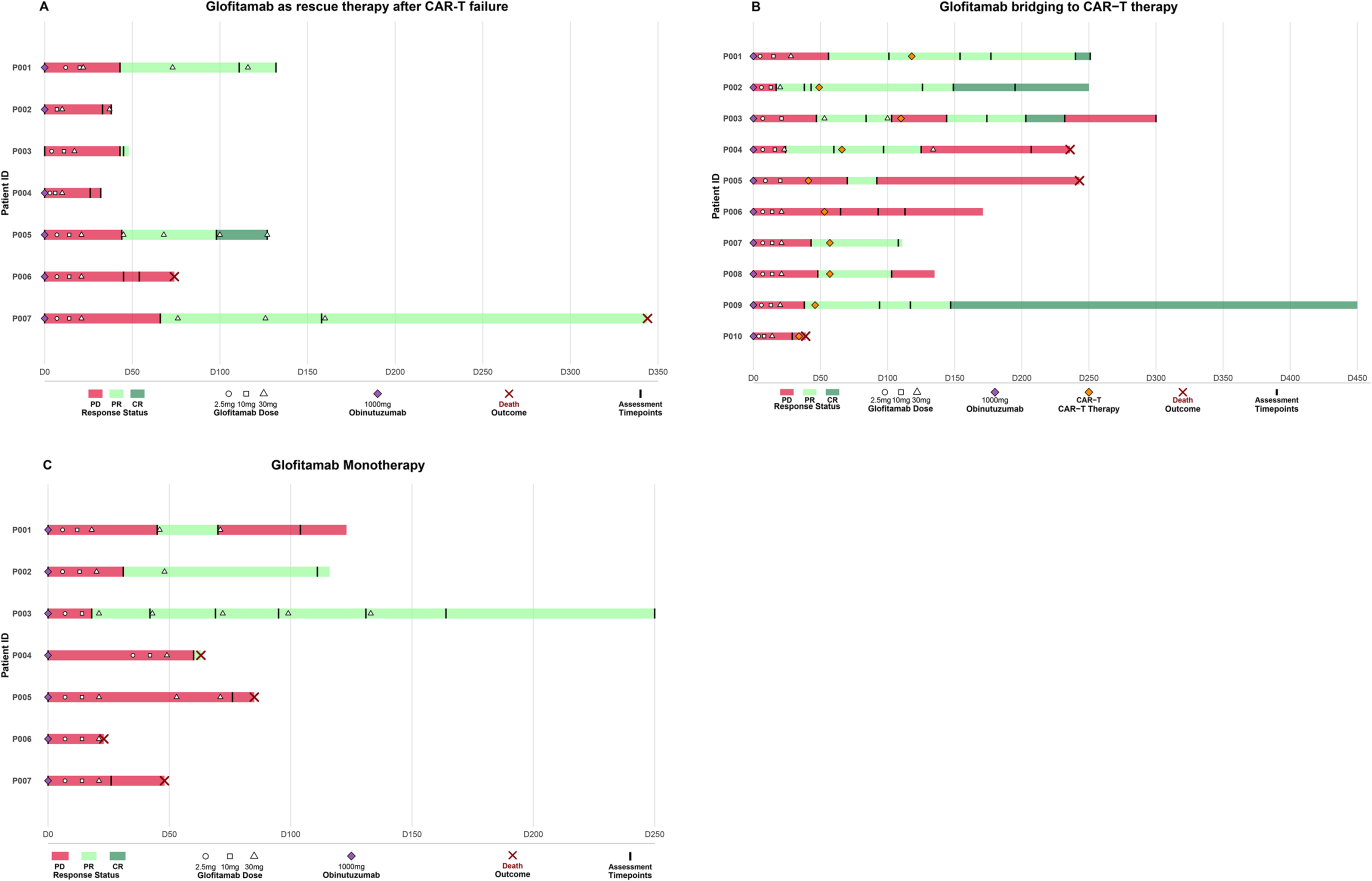
Individual patient treatment course, disease status, and terminal events in the three sequential treatment groups. **(A)** Group 1 (Glofitamab as rescue therapy after CAR-T failure, n=7). **(B)** Group 2 (Glofitamab bridging to CAR-T therapy, n=10). **(C)** Group 3 (Glofitamab monotherapy, n=7). Each horizontal bar represents one patient. Colors indicate best response: red, progressive disease (PD); dark green, complete response (CR); light green, partial response (PR). Black vertical bars, assessment timepoints. White symbols: circle, Glofitamab 2.5 mg; square, 10 mg; triangle, 30mg. Purple diamonds, obinutuzumab 1000 mg. Orange diamonds, CAR-T therapy. Red crosses, death outcome.

**Figures 1A-C** shows graphically the evolution of the disease status, the schedule and the dose of the treatment administration, and the incidence of the terminal event (death) for each individual patient from the first Obinutuzumab treatment (D0) until the data cutoff date. The median follow-up time for all patients was 125 days (95% CI: 109-278). The overall ORR was 41.7% (10/24), and the CRR was 16.7% (4/24). Among them, the ORR of group 1 (Glofitamab as a rescue therapy after CAR-T failure) was the highest, reaching 57.1%(95%CI: 18.4%-90.1%). Groups 2 and 3 of ORR was 40.0% (95% CI: 12.2%-73.8%) and 28.6% (95% CI: 3.7%-71.0%). In addition, 3 patients (3/10) in group 2 (Glofitamab bridging CAR-T therapy group) achieved CR after evaluation, with CRR of 30.0% (95%CI: 6.7%-65.2%), and CRR of 14.3%(95%CI: 0.4%-57.9%) in group 1. In group 3 (the Glofitamab monotherapy arm) no patients have the complete response, therefore in the CRR we have 0.0% (95% CI: 0.0% to 41.0%). All together the objective response rates and the complete response rates for groups 1 and 2 are markedly.

**Figure 2:**
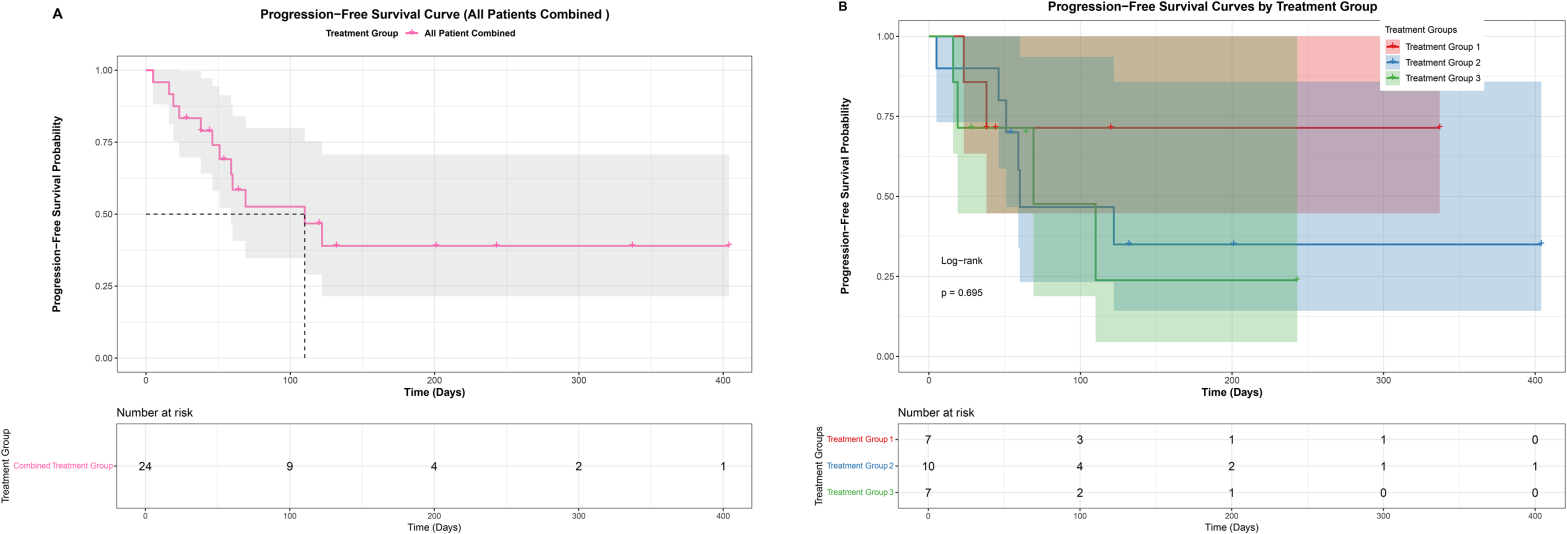
Progression free survival (PFS). **(A)** PFS for all 24 patients (median 110 days; 1 year PFS 39%, 95% CI: 21.5%. 70.7%). **(B)** PFS by treatment group. PFS was defined from CAR-T infusion for Group 2 (bridging) and from first Glofitamab dose for Groups 1 and 3. Red curve, Group 1 (salvage after CAR-T failure); blue curve, Group 2; green curve, Group 3. Log rank p=0.695.

The PFS for the whole cohort was 110 days, and the 1 year PFS was estimated at 39% (95% CI: 21.5% to 70.7%) (**Figure 2A**). Analysis of the different treatment scenarios found that in group 1, only two out of the seven patients had disease progression, therefore the survival curve is truncated at 50%. This means that more than half of the patients had stable disease during the follow-up period, and the estimated 1-year PFS was 71.4%(29.0%-100.0%). Survival outcomes were more similar in group 2 and group 3, with median PFS of 59 days and 69 days, respectively, and 1-year PFS of 40.0% (95% CI: 12.2%-73.8%) and 42.9% (95% CI: 9.8%-85.0%), respectively (**Figure 2B**). A log-rank test of PFS for the three groups did not show any difference (P = 0.695). Probably the small sample size made these estimates imprecise, which is shown by the wide confidence intervals, and the bad statistical power, that could hide any real underlying difference between the groups.

### 3.3 Dynamic changes of immune cells

**Figure 3:**
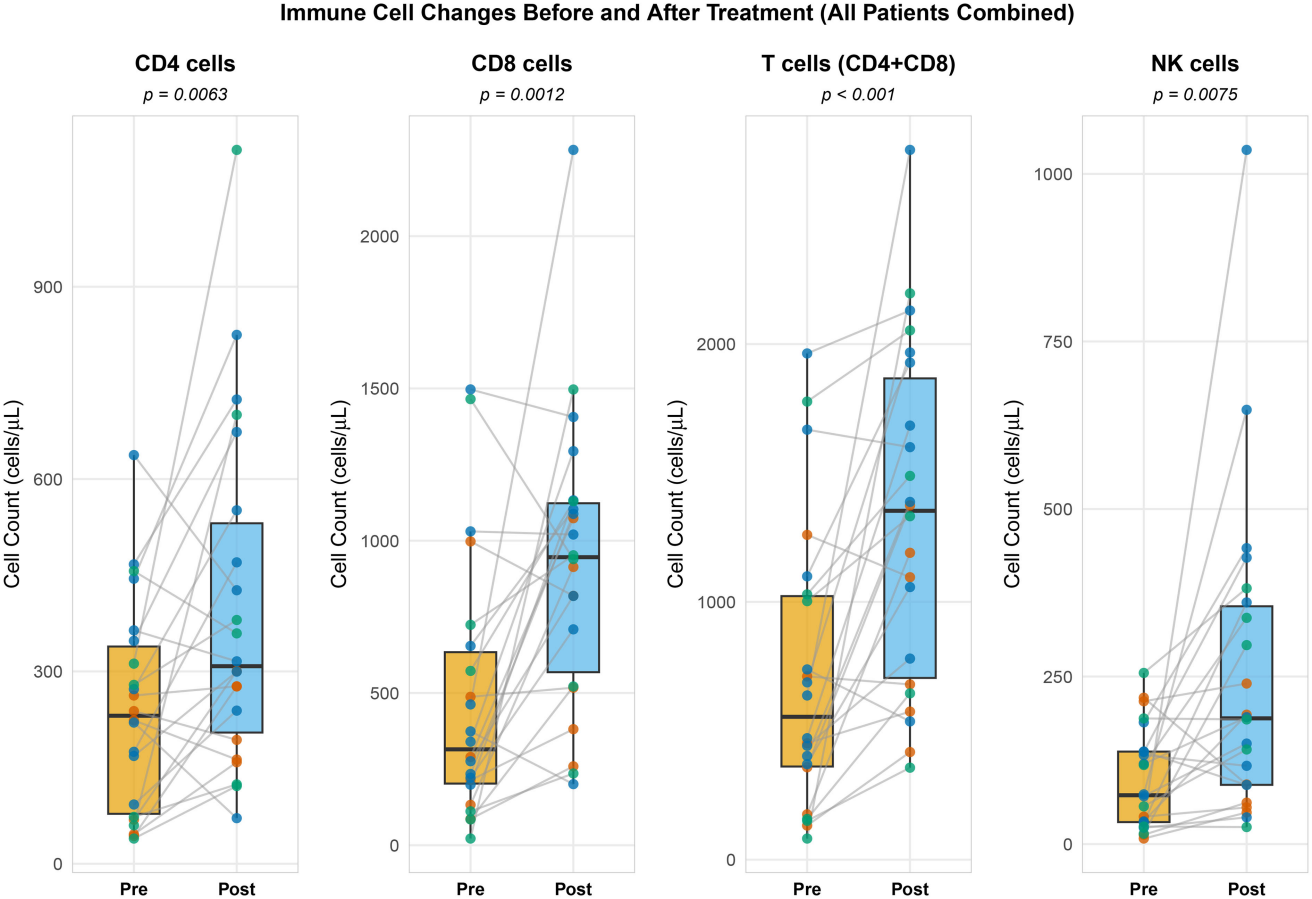
Changes in peripheral blood T cell and NK cell counts before and after Glofitamab treatment (all patients combined). Orange, pre treatment; blue, post treatment. Points colored by group (red: Group 1; blue: Group 2; green: Group 3). Glofitamab significantly increased CD8+ T cells (p=0.0063), CD4+ T cells (p=0.0012), total T cells (p<0.001), and NK cells (p=0.045).

We observed that Glofitamab treatment induced significant changes in the number of immune-cell subsets by comparing peripheral-blood samples collected before and after the first Glofitamab cycle (**Figure 3**). For all patients, the median absolute CD8+ T cell count increased from 334.4 cells/μL at baseline to 913.8 cells/μL(P = 0.0063). The median CD4+ T cell count increased from 230.9 cells/μL to 308.1 cells/μL (p=0.0012), and the median total T cell count also increased from 554.2 cells/μL to 1353.4 cells/μL (p < 0.001). In addition, the median count of NK cells (CD3-CD56+) also showed an upward trend, from 125 cells/μL to 180 cells/μL (p=0.045). These trends were generally consistent across the three treatment groups, demonstrating that the clinical efficacy of Glofitamab associated with T-cell functional activity and persistence in patients. Although NK cells are not the direct effector cells of Glofitamab, the increase of NK cells may be driven by the cytokines (such as IL-2 and IL-15) secreted by activated T cells, reflecting the broad immune activation state induced by treatment (25).

### 3.4 Safety analysis

We collected treatment-related adverse events (AEs) occurring in all patients after Glofitamab treatment and after CAR-T therapy in the Glofitamab bridging group. A total of 22 patients (91.7%) experienced at least one AE. **Table 2** details the occurrence of immunotherapy-related AEs in each treatment group. Overall, infection and hematological toxicities were the most common, followed by immune-related toxicities.

**Table 2:** Treatment-related adverse events.

Adverse event		Treatment group 1 (rescue therapy after CAR-T failure; N=7)	Treatment group 2A (glofitamab bridging phase; N=10)	Treatment group 2B (subsequent CAR-T phase; N=10)	Treatment group 3 (glofitamab monotherapy; N=7)
CRS, N(%)					
any level		4(57.1%)	8(80.0%)	6(60.0%)	4(57.1%)
grade 3-5		1(14.3%)	4(40.0%)	3(30.0%)	2(28.6%)
ICANS, N(%)					
any level		0(0%)	0(0%)	3(30.0%)	3(42.9%)
grade 3-5		0(0%)	0(0%)	2(20.0%)	1(14.3%)
IEC-HS, N(%)					
any level		1(14.3%)	2(20.0%)	2(20.0%)	2(28.6%)
grade 3-5		1(14.3%)	2(20.0%)	1(10.0%)	2(28.6%)
Anemia, N(%)					
any level		6(85.7%)	10(100.0%)	10(100.0%)	6(85.7%)
grade 3-5		3(42.9%)	3(30.0%)	6(60.0%)	4(57.1%)
Leukopenia, N(%)					
any level		4(57.1%)	8(80.0%)	7(70.0%)	6(85.7%)
grade 3-5		3(42.9%)	3(30.0%)	6(60.0%)	3(42.9%)
Thrombocytopenia, N(%)					
any level		4(57.1%)	7(70.0%)	8(80.0%)	6(85.7%)
grade 3-5		3(42.9%)	4(40.0%)	5(50.0%)	6(85.7%)
Liver dysfunction, N(%)					
any level		0(0%)	1(10.0%)	3(30.0%)	1(14.3%)
grade 3-5		0(0%)	1(10.0%)	1(10.0%)	0(0%)
Kidney dysfunction, N(%)					
any level		0(0%)	1(10.0%)	1(10.0%)	0(0%)
grade 3-5		0(0%)	0(0%)	0(0%)	0(0%)
Infection, N(%)					
any level		6(85.7%)	10(100.0%)	9(90.0%)	6(85.7%)
grade 3-5		6(85.7%)	7(70.0%)	9(90.0%)	6(85.7%)

For Group 2, events are shown separately for the Glofitamab bridging phase (2A) and the subsequent CAR-T phase (2B). Abbreviations: CRS, Cytokine release syndrome; ICANS,Immune effector cell-associated neurotoxicity syndrome;IEC-HS,Immune effector cell-associated hemophagocytic lymphohistiocytosis-Like Syndrome.

^A^ Adverse reactions related to the use of Glofitamab in Treatment group 2.

^B^ Adverse reactions related to the use of CAR-T in Treatment group 2.

#### 3.4.1 Immune-related adverse events

CRS was the most common immune-related AE, and 16 patients (66.7%) experienced varying degrees of CRS in the entire cohort.In Group 3 (monotherapy), the incidence of any-grade CRS was 57.1% (4/7), with grade 3-5 CRS occurring in 28.6% (2/7). In Group 1 (rescue after CAR-T), the CRS incidence was similar to that in Group 3 (any-grade 57.1%, grade 3-5 14.3%). Although CRS occurred in more than half of both groups, the probability of grade ≥3 CRS was low. Notably, in group 2 (bridging therapy), the Glofitamab bridging phase (group 2A, n=10) had the highest incidence of any grade CRS (80.0%), with grade 3–5 events reaching 40.0%; However, in the subsequent CAR-T phase (group 2B, n=10), the incidence of CRS decreased (any grade 60.0%, grade 3-5 30.0%) and was comparable to the incidence reported in previous studies of CAR-T monotherapy (26), and this indicates that the combination of the two treatment regimens does not necessarily increase the risk of CRS. ICANS mainly occurred in group 2 during the CAR-T treatment phase (group 2B, any grade 30.0%, grade 3-5 20.0%) and monotherapy group 3 (42.9% of any grade, 14.3% of grade 3-5), while no ICANS events were observed in group 1 or during the Glofitamab bridging phase of group 2. Although IEC-HS was rare in any group (any grade 14.3%-28.6%), all occurred as severe (grade ≥3), and given the high mortality and severe poor prognosis of IEC-HS (27, 28), it is important to closely monitor the vital signs of patients with possible IEC-HS. Unfortunately, one patient died of severe IEC-HS in group 3.

#### 3.4.2 Hematological toxicity

Among all the patients, 22 cases (91.7%) experienced hematological toxicity. Among all the hematological toxicities, anemia had the highest incidence in each group (any grade 87.5%–100%), with grade 3-5 events occurring in 30.0%–60.0% of patients. Leukopenia and thrombocytopenia were also very common, any-grade incidence was from 57.1% to 85.7%. Additionally, the incidence of grade 3–5 thrombocytopenia in the group 3(monotherapy) was as high as 85.7% (6/7), significantly higher than that of the other groups. One patient in group 2 died of a cerebral hemorrhage, which is a consequence of profound bone marrow suppression.It is worth to underline that all three groups had a considerable probability of suffering for hematological toxicity. Severe myelosuppression is not only associated with the risk of bleeding, but may also be a relevant contributor of the subsequent serious infections due to agranulocytosis (29).

#### 3.4.3 Other adverse events

Infection was also an important complication, with an incidence of 91.7% among all patients. What was particularly serious is that the incidence of severe infection was extremely high (70.0%-90.0%), which was closely related to the patients’ underlying immunocompromised status and treatment-induced severe bone marrow suppression. Among all the deceased patients, 50% (4/8) died from severe infections. Moreover, one patient in the group 3 temporarily discontinued Glofitamab due to severe pneumonia. Therefore, the monitoring and management of infections is of critical importance in clinical practice. In addition, the incidences of liver dysfunction and kidney dysfunction were relatively low, and they were mainly minor events, which is consistent with previous reports (30).

## 4 Discussion

In this single-center real-world setting, we systematically evaluated the efficacy and safety of Glofitamab in three different sequential treatment regimens, further demonstrating that Glofitamab offers an effective treatment option for r/r BCL. Especially in combination with CAR-T therapy, the ORR and CRR in the rescue group and the bridging group were superior to those in the monotherapy group. Some patients who received either CAR-T or BsAb monotherapy did not achieve long-term remission, which is consistent with the results of previous studies (31). Our study in group 1 showed that Glofitamab could achieve an ORR of 57.1% in patients who had failed CAR-T therapy. Previous studies have shown that patients who failed CAR-T treatment had extremely poor prognosis and the efficacy of traditional rescue treatment was limited (32). Furthermore, according to the research of group 2, Glofitamab was demonstrated to be a reliable option for some patients to temporarily control the progression of the disease before CAR-T infusion (17, 19).

In this study, the group 1(the group receiving Glofitamab as a rescue treatment after CAR-T therapy) had the highest 1-year PFS rate, reaching 71.4%, and more than half of the patients did not experience disease progression during the follow-up period. The PFS differences between the other two groups were not significant. Although there were numerical differences in median PFS and 1-year PFS rates among the different groups, the Log-rank test indicated that these differences were not statistically significant (P = 0.695) and the confidence intervals were wide. This might be related to the insufficient sample size and follow-up time in this study. Therefore, the long-term efficacy needs to be confirmed with larger sample sizes and extended follow-up.

By collecting peripheral blood samples of T cells and NK cells before and after the treatment cycle, we found that the median absolute count of T cells, particularly CD8+ T cells, significantly increased after treatment with Glofitamab (p < 0.001). The CD8+ T cells, as the main cytotoxic effectors, indicate that Glofitamab has mobilized the core strength of the host anti-tumor immunity. Meanwhile,the concurrent increase in CD4+ T cells may provide the necessary cytokine support and helper functions for CD8+ T cell effector activity, together constituting a coordinated adaptive immune response. This indicates that Glofitamab, through connecting T cells with tumor cells and inducing the activation and proliferation of T cells, operates on the core mechanism of inducing T cell activation and proliferation (9, 33).

In addition, we also focused on the occurrence of immunotherapy-related AEs. 91.7% of the patients experienced AEs to varying degrees. Infection was the most common AE, and the incidence of severe infection also over 70%, which not only led to the interruption of treatment plans but also resulted in the death of 16.7% (4/24) of the patients. Therefore, it is necessary to monitor the infection indicators of patients and prevent the use of antibiotics in the application of Glofitamab. Among immune-related adverse events, CRS had the highest incidence (66.7%), with severe CRS (grade ≥3) occurring in 25% of patients. Moreover, one patient died due to severe CRS. In addition, the overall incidences of ICANS and IEC-HS were low, but one patient still died from severe IEC-HS (grade ≥3). We also observed that in Group 2 (Glofitamab bridging to CAR-T therapy), the incidences of CRS and IEC-HS did not increase after CAR-T treatment, and only three patients developed new-onset ICANS, indicating that Glofitamab has no significant impact on immune-related adverse events after CAR-T treatment. This further proved the safety of Glofitamab bridging CAR-T therapy. 22 patients (91.7%) experienced hematological toxicity, with grade 3-5 hematological toxicity events occurring in 30%–60%, indicating that monitoring blood routine indicators and timely symptomatic management are also important.

This study still has some notable limitations. First, the sample size is insufficient, which leads to wide confidence intervals for the efficacy estimates and may have resulted in inadequate statistical power to detect true differences between groups, such as the trend toward superior PFS observed in group 1. Secondly, the follow-up period was relatively short (median 125 days), which limited our assessment of long-term survival outcomes, especially OS after bridging therapy. Thirdly, as a single-center retrospective study, selection bias is inevitable. Patient groups were based on clinical decision rather than randomization, and subtle differences in baseline characteristics between groups may affect the results.

This study confirmed that Glofitamab is an effective tool for r/r BCL in real-world clinical practice. However, its significant hematological toxicity and associated high infection risk require clinicians to conduct close patient management and timely supportive care. Our study included small numbers of DLBCL-RT and BL cases, which have poorer prognoses than conventional DLBCL. This heterogeneity may have biased our estimates. Sensitivity analyses excluding these subtypes showed consistent trends, but sample sizes were too small for robust subgroup conclusions.In the future, multi-center and large-sample real-world studies should be conducted to more accurately evaluate the benefits of Glofitamab in different sequential treatment strategies; a more in-depth analysis of the phenotypes of expanded T cells (e.g., effector memory cells) and the expansion curves should be carried out to identify biomarkers that predict efficacy or resistance, thereby guiding individualized treatment for patients. Future studies should explore the optimal dosage and timing of Glofitamab in combination with CAR-T therapy, optimize the treatment strategy and further enhance the therapeutic effect. In addition, future prospective multicenter studies should directly compare Glofitamab-based sequential strategies with standard-of-care options in the post-CAR-T failure and bridging settings, to better define its relative efficacy and optimal positioning.

## Data Availability

The raw clinical data supporting the conclusions of this article are not publicly available because they contain potentially identifying patient information and are subject to institutional privacy and ethics restrictions. De-identified data may be made available by the corresponding authors upon reasonable request, subject to approval by the Ethics Committee of Shanghai Tongji Hospital, Tongji University.
